# Supplementary material for: Polatuzumab vedotin combined with bendamustine and rituximab for relapsed/refractory diffuse large B-cell lymphoma: A systematic review protocol
Source: PLoS One. 2024 Aug 1;19(8):e0308247. doi: 10.1371/journal.pone.0308247 (PMC11293739; doi:10.1371/journal.pone.0308247)
Supplement: S1 Table — (DOCX) [file pone.0308247.s001.docx]

**PRISMA-P (Preferred Reporting Items for Systematic review and Meta-Analysis Protocols) 2015 checklist: recommended items to address in a systematic review protocol***

| Section and topic | Item No | Checklist item | Answer |
| --- | --- | --- | --- |
| ADMINISTRATIVE INFORMATION | | |  |
| Title: |  |  |  |
| Identification | 1a | Identify the report as a protocol of a systematic review | Yes, this protocol clearly identifies the report as a systematic review protocol |
| Update | 1b | If the protocol is for an update of a previous systematic review, identify as such | Yes, the authors expressly point out that this is not an update of a previous review. |
| Registration | 2 | If registered, provide the name of the registry (such as PROSPERO) and registration number | Yes, it is registered in the PROSPERO database, registration number provided. |
| Authors: |  |  |  |
| Contact | 3a | Provide name, institutional affiliation, e-mail address of all protocol authors; provide physical mailing address of corresponding author | Yes, names, affiliations and contact details of all authors listed. |
| Contributions | 3b | Describe contributions of protocol authors and identify the guarantor of the review | Yes, individual author contributions, described in the “Author Contributions” section. |
| Amendments | 4 | If the protocol represents an amendment of a previously completed or published protocol, identify as such and list changes; otherwise, state plan for documenting important protocol amendments | Yes, plan to document and report any protocol amendments described. |
| Support: |  |  |  |
| Sources | 5a | Indicate sources of financial or other support for the review | Yes, sources of funding (or lack thereof) are indicated. |
| Sponsor | 5b | Provide name for the review funder and/or sponsor | No sponsors involved. |
| Role of sponsor or funder | 5c | Describe roles of funder(s), sponsor(s), and/or institution(s), if any, in developing the protocol | No role for sponsors/financiers as there is no external funding. |
| INTRODUCTION | | |  |
| Rationale | 6 | Describe the rationale for the review in the context of what is already known | Indeed, an extensive justification presented within the framework of the body of current literature. |
| Objectives | 7 | Provide an explicit statement of the question(s) the review will address with reference to participants, interventions, comparators, and outcomes (PICO) | Yes, clear and unambiguous statement of the research question and PICO components |
| METHODS | | |  |
| Eligibility criteria | 8 | Specify the study characteristics (such as PICO, study design, setting, time frame) and report characteristics (such as years considered, language, publication status) to be used as criteria for eligibility for the review | Yes, in-depth requirements for eligibility were provided for research designs, subjects, comparisons, interventions, and outcomes. |
| Information sources | 9 | Describe all intended information sources (such as electronic databases, contact with study authors, trial registers or other grey literature sources) with planned dates of coverage | Yes, all intended sources of information are listed, including databases and gray literature sources. |
| Search strategy | 10 | Present draft of search strategy to be used for at least one electronic database, including planned limits, such that it could be repeated | Yes, the draft PubMed search strategy serves as an example database. |
| Study records: |  |  |  |
| Data management | 11a | Describe the mechanism(s) that will be used to manage records and data throughout the review | Yes, data management and storage process described. |
| Selection process | 11b | State the process that will be used for selecting studies (such as two independent reviewers) through each phase of the review (that is, screening, eligibility and inclusion in meta-analysis) | Yes, the study selection process will be duplicated by two independent reviewers as outlined. |
| Data collection process | 11c | Describe planned method of extracting data from reports (such as piloting forms, done independently, in duplicate), any processes for obtaining and confirming data from investigators | Yes, planned method for data extraction, including piloting forms and obtaining missing data, described. |
| Data items | 12 | List and define all variables for which data will be sought (such as PICO items, funding sources), any pre-planned data assumptions and simplifications | Yes, all variables and data elements to be extracted are clearly listed. |
| Outcomes and prioritization | 13 | List and define all outcomes for which data will be sought, including prioritization of main and additional outcomes, with rationale | Yes, primary and secondary outcomes are defined and prioritized with justification. |
| Risk of bias in individual studies | 14 | Describe anticipated methods for assessing risk of bias of individual studies, including whether this will be done at the outcome or study level, or both; state how this information will be used in data synthesis | Yes, Cochrane RoB-1 tool selected, justification and process detailed. |
| Data synthesis | 15a | Describe criteria under which study data will be quantitatively synthesised | Yes, criteria for quantitative synthesis provided. |
|  | 15b | If data are appropriate for quantitative synthesis, describe planned summary measures, methods of handling data and methods of combining data from studies, including any planned exploration of consistency (such as I^2^, Kendall’s τ) | Yes, planned summary measures, statistical models and methodologies provided. |
|  | 15c | Describe any proposed additional analyses (such as sensitivity or subgroup analyses, meta-regression) | Yes, additional analyses such as subgroup, meta-regression and sensitivity analyses are described. |
|  | 15d | If quantitative synthesis is not appropriate, describe the type of summary planned | Yes, if meta-analysis is not appropriate, plan a qualitative synthesis, described in the “Qualitative Synthesis” section. |
| Meta-bias(es) | 16 | Specify any planned assessment of meta-bias(es) (such as publication bias across studies, selective reporting within studies) | Yes, methods for assessing possible meta-biases such as Publication bias is described. |
| Confidence in cumulative evidence | 17 | Describe how the strength of the body of evidence will be assessed (such as GRADE) | Yes, the GRADE method will be used to assess the quality of the evidence in depth. |

*** It is strongly recommended that this checklist be read in conjunction with the PRISMA-P Explanation and Elaboration (cite when available) for important clarification on the items. Amendments to a review protocol should be tracked and dated. The copyright for PRISMA-P (including checklist) is held by the PRISMA-P Group and is distributed under a Creative Commons Attribution Licence 4.0.**

*From: Shamseer L, Moher D, Clarke M, Ghersi D, Liberati A, Petticrew M, Shekelle P, Stewart L, PRISMA-P Group. Preferred reporting items for systematic review and meta-analysis protocols (PRISMA-P) 2015: elaboration and explanation. BMJ. 2015 Jan 2;349(jan02 1):g7647.*
