# Supplementary material for: Polatuzumab vedotin combined with bendamustine and rituximab for relapsed/refractory diffuse large B-cell lymphoma: A systematic review protocol
Source: PLoS One. 2024 Aug 1;19(8):e0308247. doi: 10.1371/journal.pone.0308247 (PMC11293739; doi:10.1371/journal.pone.0308247)
Supplement: Attachment — Submitted filename: Comments for Polatuzumab vedotin.docx [file pone.0308247.s002.docx]

The background section of this manuscript effectively sets the stage by highlighting the clinical significance of diffuse large B-cell lymphoma (DLBCL) and the challenges associated with relapsed/refractory cases. The introduction of Polatuzumab vedotin combined with bendamustine and rituximab (Pola-BR) as a promising salvage therapy is well-articulated. However, the Abstract paragraph could benefit from briefly mentioning previous standard-of-care treatments and how Pola-BR compares in terms of mechanisms of action or preliminary efficacy. Additionally, providing more context on the prevalence and impact of DLBCL would strengthen the justification for this systematic review.

The authors mention the expected lack of appropriate studies for conducting subgroup analyses. Can they specify any pre-defined subgroups they intend to analyze and the criteria for these subgroups?

Given the novelty of Pola-BR, how do the authors plan to address the potential lack of data on long-term outcomes and safety? Will there be any focus on collecting or analyzing data from ongoing or upcoming trials?

The manuscript mentions the use of the ROB-1 risk of bias assessment tool. Are there any additional tools or methods the authors will employ to ensure a thorough assessment of study quality and bias?

How do the authors envision the findings of this systematic review influencing current clinical guidelines and decision-making processes for treating R/R DLBCL?

Can the authors provide more detailed examples of the research gaps they expect to identify, and how these might direct future studies or therapeutic development?

The protocol claims to include literature without language restrictions. What specific measures will be taken to ensure accurate translation and interpretation of non-English studies? How do the authors plan to address potential publication bias, especially considering the novelty of Pola-BR and the possibility of unpublished negative results?

The background mentions the conventional R-CHOP therapy and its outcomes. Are there any recent advancements or modifications to this protocol that could be relevant to the study? Can the authors elaborate on the mechanisms of action for bendamustine and rituximab and how they synergize with polatuzumab vedotin in the Pola-BR regimen?

The background references a phase 1b/2 trial (GO29365) showing promising results for Pola-BR. Can the authors provide more specifics about the trial’s design, sample size, and key findings?

The manuscript states that overall survival (OS) is the primary outcome measure. Why was OS chosen over other potential primary endpoints, such as progression-free survival (PFS) or overall response rate (ORR)? How do the authors plan to handle and interpret potential discrepancies between OS and PFS results? Besides, correct the typo in this sentence “The preferred reporting items for systemic reviews and 6 meta-analysis protocols (PRISMA-P) will be followed in the review process”.
