# Supplementary material for: Polatuzumab vedotin combined with bendamustine and rituximab for relapsed/refractory diffuse large B-cell lymphoma: A systematic review protocol
Source: PLoS One. 2024 Aug 1;19(8):e0308247. doi: 10.1371/journal.pone.0308247 (PMC11293739; doi:10.1371/journal.pone.0308247)
Supplement: Attachment — Submitted filename: For the Academic editor.docx [file pone.0308247.s003.docx]

Dear Dr. Chen Li,

I hope this letter finds you well. I am writing to inform you about the modifications made to our manuscript based on the templates provided.

We carefully checked and updated our manuscript based on the templates. In particular, we added an appropriate caption for the supporting information at the end of the manuscript file, which includes the completed PRISMA-P checklist.

The caption for the PRISMA-P checklist is now:

**S1** **Table.** Completed checklist “Preferred Reporting Items for Systematic Review and Meta-Analysis Protocols (PRISMA-P) 2015”.

Thank you for your time and consideration. We look forward to your feedback and the next steps in the review process.

Sincerely,

Mohammadreza Eslami
